# Experimental Study of Nasopharyngeal Carcinoma Radionuclide Imaging and Therapy Using Transferred Human Sodium/Iodide Symporter Gene

**DOI:** 10.1371/journal.pone.0117053

**Published:** 2015-01-23

**Authors:** Xing Zhong, Changzheng Shi, Jian Gong, Bin Guo, Mingzhu Li, Hao Xu

**Affiliations:** 1 Department of Medical Imaging Center, First Affiliated Hospital, Jinan University, Guangzhou 510630, China; 2 Department of Nuclear Medicine, First Affiliated Hospital, Jinan University, Guangzhou 510630, China; Northwestern University Feinberg School of Medicine, United States of America

## Abstract

**Purpose:**

The aim of this study was to design a method of radionuclide for imaging and therapy of nasopharyngeal carcinoma (NPC) using the transferred human sodium/iodide symporter (hNIS) gene.

**Methods:**

A stable NPC cell line expressing hNIS was established (CNE-2-hNIS). After ^131^I treatment, we detected proliferation and apoptosis of NPC cells, both in vitro and vivo. In vivo, the radioactivity of different organs of nude mice was counted and ^99m^Tc imaging using SPECT was performed. The apparent diffusion coefficient (ADC) value changes of tumor xenografts were observed by diffusion-weighted magnetic resonance imaging (DW-MRI) within 6–24 days of ^131^I treatment. The correlation of ADC changes with apoptosis and proliferation was investigated. Post-treatment expression levels of P53, Bax, Bcl-2, Caspase-3, and Survivin proteins were detected by western blotting.

**Results:**

^131^I uptake was higher in CNE-2-hNIS than in CNE-2 cells. The proliferation and apoptosis rate decreased and increased respectively both in vitro and vivo in the experimental group after ^131^I treatment. The experimental group tumors accumulated ^99m^Tc in vivo, leading to a good visualization by SPECT. DW-MRI showed that ADC values increased in the experimental group 6 days after treatment, while ADC values were positively and negatively correlated with the apoptotic and Ki-67 proliferation indices, respectively. After treatment, CNE-2-hNIS cells up-regulated the expression of P53 and Survivin proteins and activated Caspase-3, and down-regulated the expression of Bcl-2 proteins.

**Conclusions:**

The radionuclide imaging and therapy technique for NPC hNIS-transfected cell lines can provide a new therapy strategy for monitoring and treatment of NPC.

## Introduction

Nasopharyngeal carcinoma (NPC) is one of the most common malignant tumors in Southern and Southeast Asia, with an annual incidence of 10–30 per 100,000 people. Although NPC is distinctly radiosensitive, it has a high rate of treatment failure because of its metastatic behaviour and locoregional recurrence [1, 2].

NIS is an integral membrane protein located on the basolateral surface of the thyroid follicular cells that mediate the uptake and concentration of iodide into the thyroid gland [3, 4]. NIS gene transfection offered a new approach to treatment of extrathyroidal malignancies. While cloning of the NIS gene can be transfected into different tumor cell lines, the uptake of radioactive iodide should partially inhibit cells’ growth. ^131^I targeted therapy mediated by transfected NIS uses the β-rays emitted during ^131^I decay. Differences in the retention amount and time of ^131^I in the cells will lead to different biological effects. Currently, the exact biological mechanism to achieve ^131^I targeted therapy in NIS-transfected non-thyroid cancer has not been established.

As previously shown [5], the clinical evaluation of tumor treatment is to measure tumor volume and morphological changes. However, therapeutic-induced changes in tumor volume often occur relatively late during the treatment. Sometimes, new therapy methods may not lead to a significant reduction in tumor size, yet it can lead to a variety of biological effects, including inhibition of growth or apoptotic-induced cell death. There is a need to evaluate new therapeutic methods that will reflect changes at the cellular level. Recently, it was shown that diffusion-weighted magnetic resonance imaging (DW-MRI) can be used as a cancer biomarker, highlighting the potential of this promising technique in evaluating early treatment response in cancer patients [6]. DW-MRI can provide microstructural information at the cellular level. Both pre-clinical [7, 8] and clinical studies [9] have revealed that treatment success in a wide variety of tumor types can be detected as an increase in their apparent diffusion coefficient (ADC) values.

The aim of our study was to investigate in the experiment environment the potential of radioiodine imaging and treatment of NPC cells after transfection with the hNIS gene and to evaluate early response to treatment using DW-MRI, and the response of ^131^I treatment of NPC from protein level were initially explored.

## Materials and Methods

In our study, a stable NPC cell line expressing hNIS was firstly established, and then the kinetics of ^125^I and the toxic effects of ^131^I in CNE-2-hNIS were observed. There were exciting results in vitro, so we proceeded to in vivo studies.

### Plasmid constructs and establishment of stably transfected CNE-hNIS cell lines

NPC cell line CNE-2 was purchased from the American Type Cell Collection (ATCC). The Cells were cultured in RPMI-1640 medium (Gibco) supplemented with 10% fetal calf serum (Gibco) in an incubator (5% CO_2_, 37°C), and the medium was freshed every 2–3 days. The cells were harvested with 0.25% trypsin (Gibco) when needed.

hNIS cDNA was amplified from a human thyroid cDNA bank, and was cloned into plasmid pCMV-Tag2 (Invitrogen, Karlsruhe, Germany) to get pCMV-Tag2-hNIS.

CNE-2 cells were transfected with pCMV-Tag2-hNIS by LipofectAMINE 2000，stable clones were selected by addition of 500μg/ml geneticin(Gibco) to the medium 2 days after transfection and was named CNE-2-hNIS.

### Demonstration of hNIS protein expression in CNE-hNIS

The CNE-2 and CNE-2-hNIS cells were lysed in RIPA buffer with standard protease inhibitors (Santa Cruz Biotechnology, Santa Cruz, CA) and standard Western blotting analyses were performed as described previously [10] using primary antibodies hNIS (abcam).

### Kinetics of ^125^I uptake

Uptake of ^125^I was determined as described for iodide by Weiss et al [11]. In brief, approximately 3×10^5^ cells were incubated with Hank’s buffered salt solution (HBSS) supplemented with 10μM NaI, 0.1μCi Na^125^I/ml, and 10 mM HEPES (pH 7.3) with and without 1 M perchlorate. After 60 min they were washed twice with ice-cold HBSS, lysed in 1 M NaOH, and counted using a Cobra II auto-gamma gamma counter. For the internalization studies, 3×10^5^ cells were incubated with HBSS supplemented with 10μM NaI, 0.1μCi Na^125^I/ml, and 10 mM HEPES (pH 7.3) at 37°C for 5, 10, 15, 20, 30, 40, 60 min, respectively. Efflux of ^125^I was investigated by incubating 3×10^5^ cells with HBSS supplemented with 10μM NaI, 0.1μCi Na ^125^I/ml, and 10 mM HEPES (pH 7.3) for 1 h. Then the cells were washed twice with ice-cold HBSS, and incubated with nonradioactive HBSS for 5, 10, 20, 30 and 40 min, respectively before lysis with NaOH.

### The toxic effects of ^131^I in vitro

The CNE-2 and CNE-2-hNIS cells were first incubated in HBSS solution for 7 h, which contained 0, 100, 200, 300μCi/ml Na^131^I, respectively. Medium was changed several times in the period of 2 h to remove the excessive remaining ^131^I. The cells were then detected by cell counting kit-8 (CCK-8) at 1 to 6 day and the annexin V/PI kit at 1 to 3 day after ^131^I incubation according to the manufacturer’s instructions, and Clonogenic Assays were performed as described previously [12].

### Animal experiments

The animals were purchased from Medical Laboratory Animal Center of Guangdong (certificate number: SCXK2008–0002). This study was carried out in strict accordance with the recommendations in the Guide for the Care and Use of Laboratory Animals of the National Institutes of Health. The protocol was approved by the Committee on the Ethics of Animal Experiments of Jinan University. The sacrifice of all animals was performed under sodium pentobarbital anesthesia, and all efforts were made to minimize suffering. The humane endpoint would be applied when the xenograft tumors had reached ~30 mm in maximum diameter.

Athymic nude (BALB/C nu/nu) mice at 6–8 weeks of age were obtained from the medical laboratory animal centre comparative medical laboratory, Guangdong, China. The animals were housed under pathogen-free conditions with a 12-h light/12-h dark schedule and fed autoclaved standard food and water ad libitum. For xenografts, 6×10^6^ cells in culture medium were subcutaneously injected into the flanks of the mice.

### Radionuclide uptake studies in vivo

After subcutaneous injection of cells for six to eight weeks, xenograft tumors were grown at ~10 mm in diameter, mice (n = 5) were injected with 0.2 mCi/0.15mL ^99m^TcO4- into peritoneal cavity, mice were placed in a spread prone position and scanned with SPECT radionuclide body imaging (GE, HELIX APEX SPX) at 0.5, 1, 1.5, 2,4h after injection.

For biodistribution experiments, mice (n = 15) were killed, and then 6μCi Na^125^I was injected i.p at 0.5, 1, 2, 6, and 24h. Organs of interest were dissected and weighed, and radionuclide uptake was measured in a gamma counter. The results were reported as percentage of injected dose per gram tissue (% ID/g).

### Radionuclide therapy studies in vivo

To establish tumor models, CNE-2 or CNE-2-hNIS cells were injected s.c. into left flank of the mice respectively. When tumor diameters reached approximately 10 mm, mice (n = 30) were administered i.p 111 MBq (3 mCi) of Na^131^I (experiment group) or PBS(control group). MRI scans were performed in all mice before (baseline) and 6、12、18 and 24 days after the above-mentioned treatment.

### MR imaging and analysis

MRI was performed at a 1.5T MR unit (Sigma, General Electric Medical Systems, USA) with a maximum gradient strength of 40mT/m, which was equipped with a 3-inch surface Coil. The axial images were obtained with a section thickness of 3.0mm and an intersection gap of 0.2mm and field of view 6.0 cm×6.0 cm for T1WI and T2WI. For T2WI: repetition time(TR)/echo time (TE) of 5120/85ms; field of view(FOV) of 160×160mm; imaging acquisition matrix of 256×192. For DWI：TR/ TE of 1500/100 ms，matrix of 128×128，NEX of 4，b factors(0 and 400 sec/mm^2^) in three orthogonal gradient directions(*X*, *Y*, and *Z*).

The acquired images were sent to a dedicated workstation (Advantage Workstation, ADW4.5, GE Medical Systems) and analyzed by two experienced radiologists. Xenografts volume was calculated with the following formula: V (mm3) = L × W2 × 0.52, where L is the maximum length and W is the maximum width of the xenografts. The ADC measurement of xenografts was selected in a maximum cross-section of xenografts. Using an operator-defined region of interest (ROI), the area of the tumor was manually delineated on maximum tumor-containing slice for DWI. We measured ADC values and change rate of ADC values at different time points in the experimental group and the control group. The rate change of ADC value = (ADC values ​​after treatment—pre-treatment ADC value) / pre-treatment ADC values.

### Immunohistochemistry of Tumor Xenografts

Cells of tumor Xenografts before and 6 days after ^131^I treatment were examined for TUNEL (Roche, USA) and expression of Ki-67 and activity of Caspase-3 (Santa Cruz Biotec Inc., USA) according to the manufacturer’s instructions. Criteria for positivity of apoptotic cells, Caspase-3 activity, and Ki-67 were used as described previously [13].

### The apoptosis proteins were detected by western blotting

Xenografts at day 6 after treated with 3 mCi Na^131^I were lysed in RIPA buffer with standard protease inhibitors (Santa Cruz Biotechnology, Santa Cruz, CA) and standard Western blotting analyses were performed as described previously [10] using primary antibodies, including Bcl-2, Caspase-3(Santa Cruz, USA), Bax, P53, Surviving (CST, USA).

### Statistical Analysis

Experiments for in vitro were carried out in triplicates. For the clonogenic assay, ten wells were evaluated for the same conditions and cell density. Results are presented as means ± SD of triplicates. Statistical significance was tested by Student’s t test. Values of *p*<0.05 were used to indicate statistical significance. A linear regression method was used to describe the relationship between ADC change within tumor and apoptotic cell density or the density of cells showing Ki-67 and Caspase-3 expression.

## Results

### Iodide accumulation in CNE-2-hNIS cells

**Figure 1:**
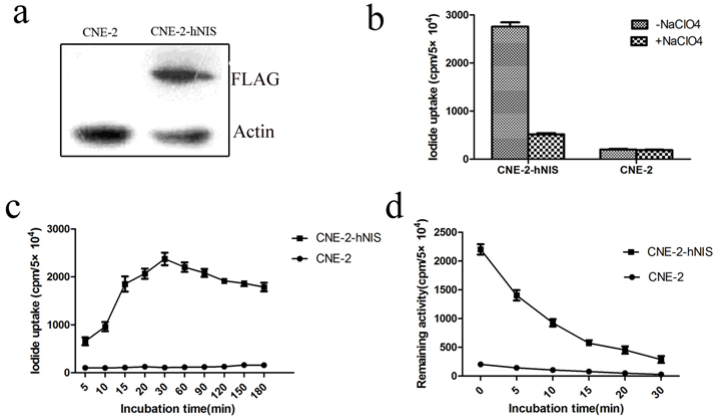
Iodide accumulation in CNE-2-hNIS cells. (a) Western blot of membrane preparations of NIS-transfected cell lines. Lanes from left to right are: CNE-2 and CNE-2-hNIS. (b) Uptake of ^125^I was significantly higher in CNE-2-hNIS cells than in CNE-2 cells (*t* = 50.5，*P*＜0.05). Uptake inhibition by perchlorate was highly significant (*t* = 42.3，*P*＜0.05). (c) Internalization of iodide into CNE-2-hNIS and CNE-2 cells. (d) Iodide efflux from CNE-2-hNIS and CNE-2 cells after 1 h incubation with Na^125^I. Data are expressed as mean±SD (n = 3).

First, we detected that CNE-2 cells did not express hNIS protein. Subsequently, we constructed a eukaryotic expression plasmid pCMV-Tag2-hNIS. After liposome-mediated transfection of CNE-2 cells with the pCMV-Tag2-hNIS plasmid, the stable CNE-2-hNIS transfected cell line was established by geneticin selection, and the expression of hNIS in cells was detected by hNIS monoclonal antibody (Fig. 1a). CNE-2-hNIS cells accumulated high amounts of radioiodide, which were completely inhibited by the addition of perchlorate, whereas the control CNE-2 cell line did not (Fig. 1b). Uptake of ^125^I was 17.1 ± 2.3-fold higher in CNE-2-hNIS cells than in CNE-2 cells. Accumulation was rapid in CNE-2-hNIS cells, reaching maximal levels within 30 min (Fig. 1c). The efflux of ^125^I from CNE-2-hNIS cells was rapid, with half-maximal activity levels reached after 8.45 min (Fig. 1d).

### The toxic effects of ^131^I in vitro

**Figure 2:**
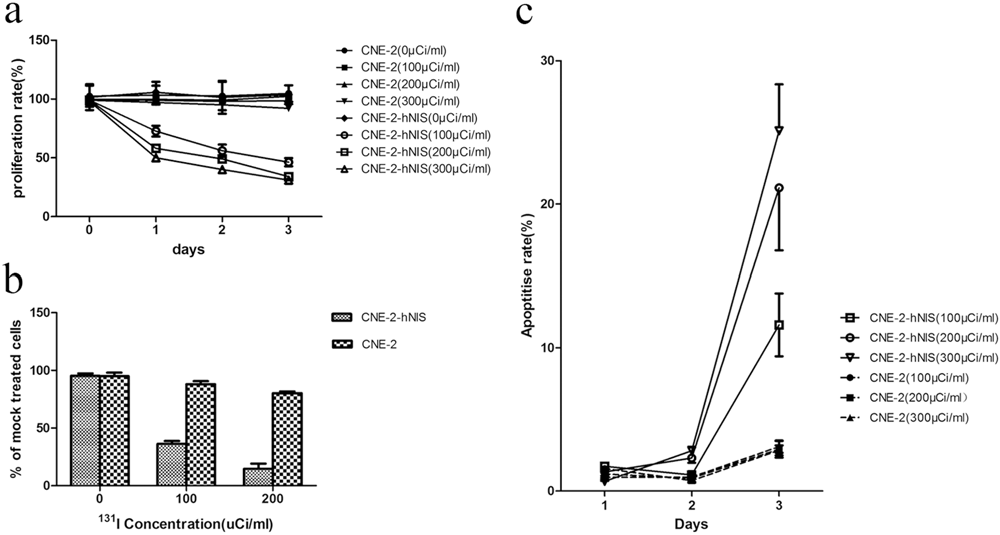
The toxic effects of ^131^I in vitro. (a) Assessment of cell proliferation in 0 to 3 days by CCK-8 of CNE-2-hNIS and CNE-2 cells after 7 h of incubation with 100 to 300 μCi/ml Na^131^I. Results are expressed as mean and SD of at least three independent determinations. (b) Clone formation of CNE-2-hNIS and CNE-2 cells in the in vitro clonogenic assay after 7 h of incubation with 0 to 200μCi/ml Na^131^I. For ease of comparison, values are depicted as percent of mock-treated cells. (c) Assessment of cell apoptosis in 0 to 3 days by Annexin V-PI of CNE-2-hNIS and CNE-2 cells after 7 h of incubation with 100–300μCi/ml Na^131^I.

First, cell proliferation was measured using CCK-8 when different concentrations of ^131^I were applied for treatment. The proliferation rates of CNE-2-hNIS gradually declined compared to those of CNE-2 at 24 and 48h after incubation with ^131^I in three concentrations and the difference was statistically significant. The proliferation of CNE-2-hNIS cells gradually decreased when the concentration of ^131^I added increased (Fig. 2a).

After treatment with 0, 100, or 200 μCi/mL of Na^131^I for 7 h, the clonogenic survival rates of CNE-2-hNIS cells were markedly reduced, in a dose-dependent manner, to 95.4% ± 2.08%, 36.33% ± 2.51%, and 14.66% ± 4.50%, respectively. The survival rates of CNE-2 cells were 95.2% ± 3.43%, 88.0% ± 2.64%, and 80.3% ± 1.52%, respectively (Fig. 2b).

After incubation with 0, 100, 200, and 300 μCi/mL of Na^131^I for 7 h, cells were assessed visually for the presence of cytotoxic effects. Annexin V-FITC/PI double staining flow cytometry experiments showed that the rate of apoptosis of CNE-2-hNIS cells increased with increasing ^131^I concentration at the 3th day (Fig. 2c).

### Biodistribution of Na^125^I in xenografts -bearing nude mice

**Figure 3:**
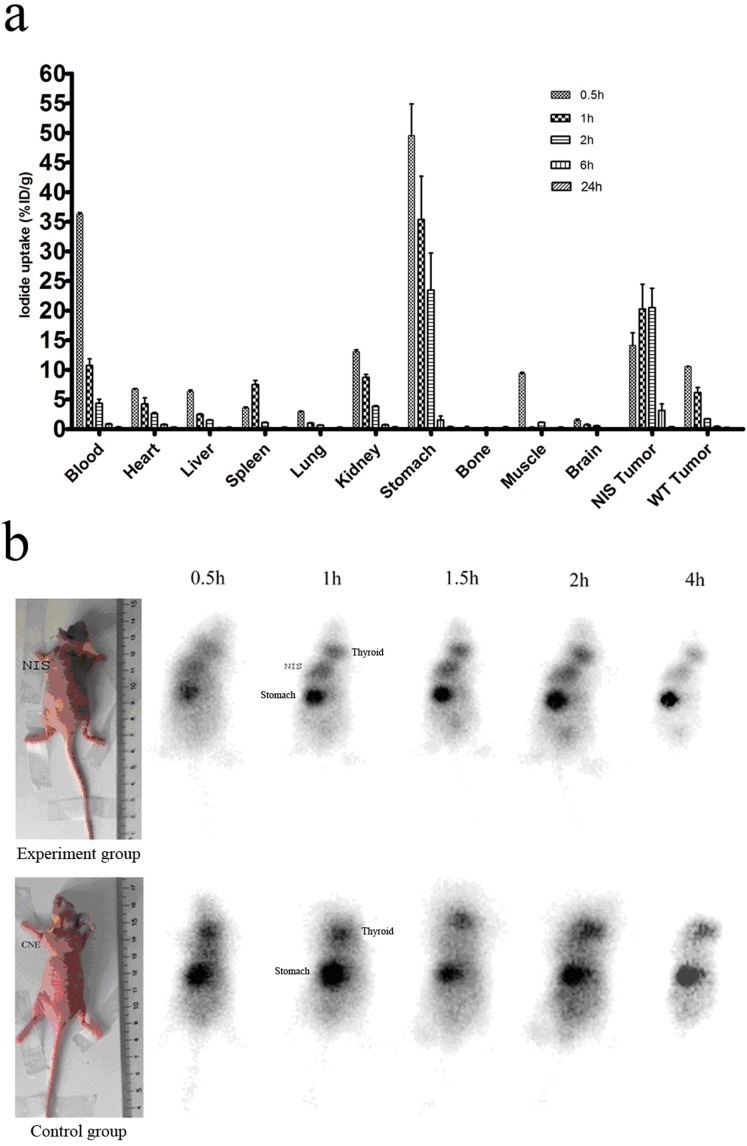
Biodistribution of Na^125^I and scintigraphic images of hNIS expression. (a) Biodistribution of radiotracer at different times after intraperitoneal administration of Na^125^I in mice bearing CNE-2-hNIS and CNE-2 cells. Data are expressed as mean%ID/g±SD(n = 3). (b) Scintigraphic images of hNIS expression. After injecting with 0.2 mCi/0.15mL ^99m^TcO4^-^ per mouse, animals were scanned for 1 min using a gamma camera from 0.5h to 4h post ^99m^Tc injection.

Results of intraperitoneal treatment of Na^125^I for CNE-2-hNIS and CNE-2 bearing nude mice were shown in biodistribution data (Fig. 3a). Compared with parental CNE-2 tumors, the NIS-expressing tumors exhibited an increased uptake of ^125^I. After tracer administration, the quantitation of the Na^125^I uptake (%ID/g) in the tumors at 1, 2, 6, and 24 h were 20.28% ± 0.15%, 18.52% ± 0.26%, 3.15% ± 0.11% and 0.04% ± 0.01%, respectively, which were significantly higher than those for the CNE-2 tumors (P < 0.05). In CNE-2-hNIS tumors, the 10.71-fold higher iodide accumulation was detected after 2 h administration when it was compared with CNE-2 tumors. However, the radioactivity remained quite stable in the CNE-2-hNIS tumors for 6 h administration, following a decline during the period until 24 h. In this model, the effective half-life (T1/2) was 1.56 h.

### ^99m^Tc imaging of xenografts

^99m^Tc scintigraphy was used to visualize the hNIS-expressing tumor in vivo. Images were taken after intraperitoneal injection of 11.1 MBq of ^99m^TcO_4_^-^ at 30, 60, 90, 120, and 240 min. The hNIS-transfected tumors were clearly visible, with intensity levels comparable to that of the thyroid. In contrast, the control tumor was not seen. Normal NIS-expressing tissues, including those of the salivary gland, thyroid gland, and stomach, and the ones involved in iodide elimination (bladder) were also clearly visible (Fig. 3b).

### Apoptotic and proliferation changes in xenografts in vivo after ^131^I treatment

**Figure 4:**
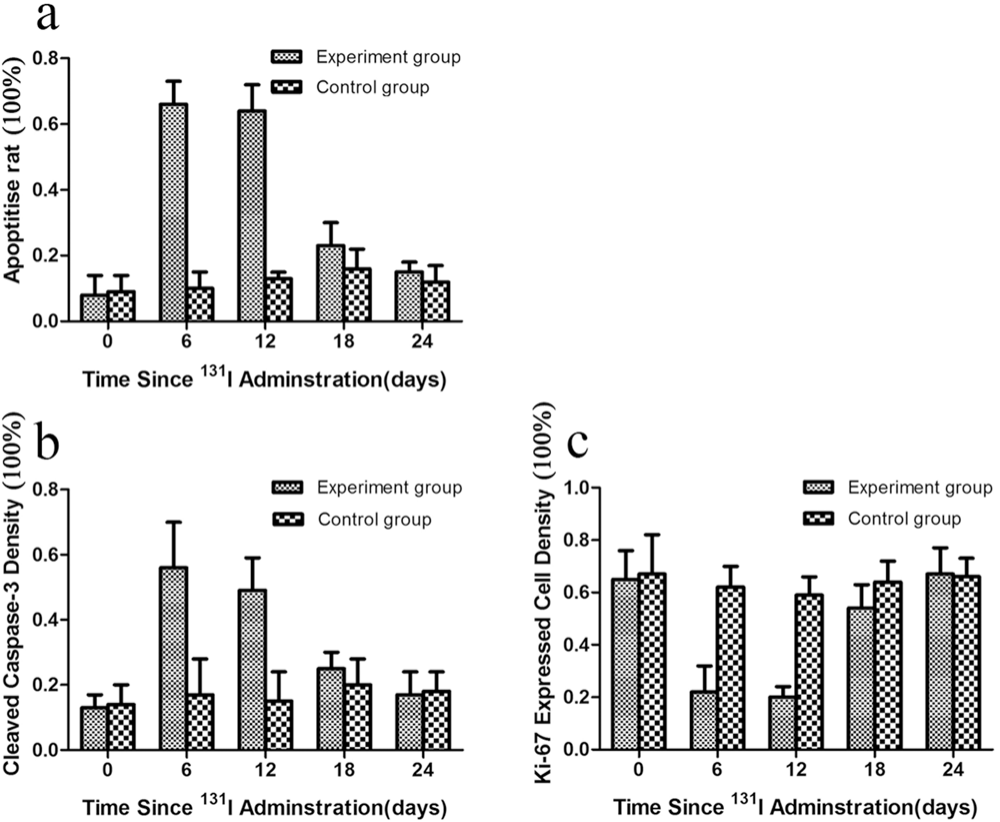
The graphs show apoptotic and proliferating cell density. The graphs show apoptotic cell density (a), cleaved caspase-3 density(b), and proliferating(Ki-67 expression) cell density(c) in xenografts at 0–24 days after ^131^I treatment in experiment group and control group.

After ^131^I treatment, the rate of apoptosis and caspase-3 positivity was higher in the experimental group than in the control group, whereas the positive rate of Ki-67 was lower at day 6 and 12 after ^131^I treatment. Overall, there was a statistically significant difference between the experimental and control groups in the rate of apoptosis, caspase-3, and Ki-67 expression at day 6 and 12 after ^131^I treatment (Fig. 4a-c).

### In vivo effect of ^131^I therapy demonstrated by MRI

**Figure 5:**
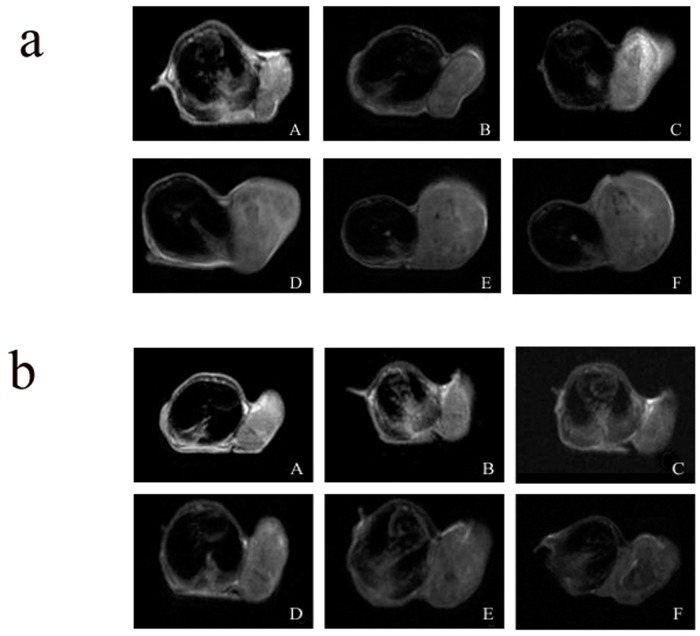
The T2WI of tumor xenografts in control group (a) and in experiment group (b) (A-F is represent respectively the T2WI of tumor xenografts before treatment, the day 6, 12,18,24 after ^131^I treatment. The tumor volumes of control xenografts rapidly increased, and the tumor volumes of the experimental xenografts were slightly larger after ^131^I treatment.).

**Figure 6:**
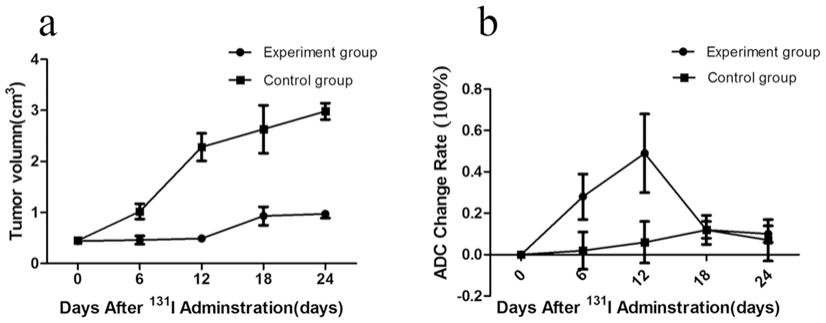
The volume change (a) and ADC change rate (b) of tumor xenografts in experiment and control group after ^131^I administration. The MRI result showed that the growth of experimental xenografts was delayed significantly compared to that of control group after ^131^I treatment. There was a statistically significant difference in ADC values between the experimental and control groups at day 6, and 12 after ^131^I treatment.

The MRI result showed that the growth of experimental xenografts was delayed significantly after ^131^I treatment. On the contrary, the size of control xenografts rapidly increased. The tumor volumes in the experimental group were slightly larger after ^131^I treatment (Fig. 5, Fig. 6a).

The ADC values of xenografts between the control and experimental groups before ^131^I therapy did not differ statistically. At day 6 after treatment, however, the ADC value increased in the experimental group, and continued to increase with time. At day 12, the ADC value reached a peak and started to decrease at day 18 and 24 after treatment. There was a statistically significant difference in ADC values between the experimental and control groups at day 6, and 12 after ^131^I treatment (Fig. 6b).

We assessed the relationship between the ADC value and the apoptosis rate by TUNEL, expression of Caspase-3, and Ki-67 after ^131^I treatment. The Pearson’s product-moment correlation coefficients were 0.72 (P <0.001), 0.65 (P = 0.001) and-0.71 (P <0.001) respectively.

### Expression pattern of apoptosis-responsive proteins after ^131^I treatment

**Figure 7:**
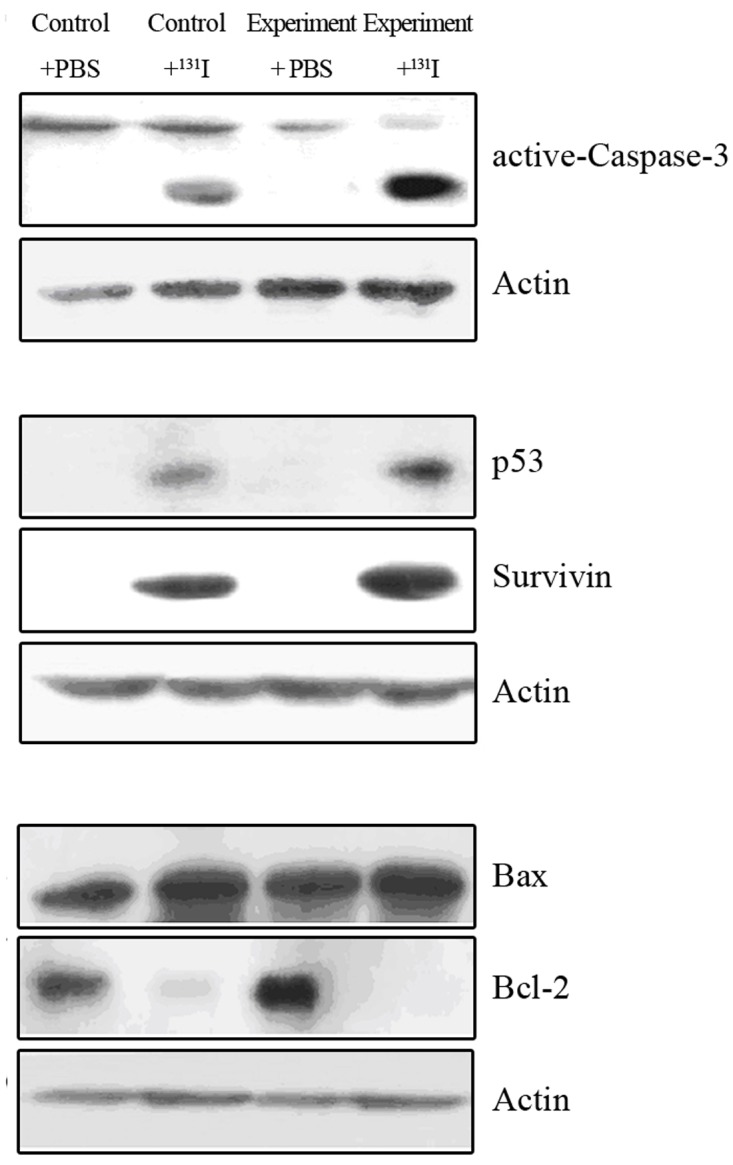
Expression of apoptosis-related proteins in CNE-2-hNIS and CNE-2 xenografts after ^131^I treatment. After ^131^I treatment, the levels of P53, activated Caspase-3, and Survivin proteins were increased, but Bcl-2 protein was decreased in CNE-2-hNIS cells compared to CNE-2 cells.

To determine which apoptosis-related proteins are regulated by ^131^I radiation, the expression of P53, Bcl-2, Bax, Caspase-3, and Survivin proteins was measured at day 6 after 3 mCi Na^131^I treatment in mice xenografts using western blot. After ^131^I treatment in NPC hNIS-transfected cells, the levels of P53, activated Caspase-3, and Survivin proteins were increased, but Bcl-2 protein was decreased compared to the control group. However, the expression of Bax did not show any change in either the experimental or the control group. This result implies that apoptosis by ^131^I may be mediated by P53, Caspase-3, Bcl-2, and the Survivin pathway in NPC hNIS-transfected cells (Fig. 7).

## Discussion

There has been reported that the attempts to induce iodide uptake by hNIS gene transfer in various human cancer cell lines, including glioma, melanoma, liver, lung, colon, ovarian, cervix, prostate, and mammary gland [14–19]. Expression of functionally active hNIS in the tumor can allow to the accumulation of radioactive iodide and then make it susceptible to radioiodide treatment. Because of the limitations of current gene therapy technology, it is difficult to achieve 100% transfection rates with the target gene. Other studies [20–22] used gene therapy to produce a “bystander” effect, so that tumor cells, which did not carry the gene could also be killed. The range of β-particles emitted by ^131^I is several millimetres, so surrounding untransfected tumor cells are likely to get damaged.

NPC is one of the most common cancers in China, and the main reason for treatment failure continues to be local recurrence and distant metastasis. Therefore, new treatment strategies are needed. We investigated the potential of radioiodide treatment in an NPC cell line (CNE-2) after hNIS transfection. Results showed rapid internalization of ^131^I into CNE-2-hNIS cells, with the maximal levels of uptake being reached in 30 min. However, ^131^I efflux from CNE-2-hNIS cells was rapid, with an effective t1/2 of 8.45 min. In our study, the retention time of iodine in vivo was longer than that in vitro. The biological half-life of ^125^I in hNIS xenografts is approximately 1.56 h. In our biodistribution study, hNIS-expressing tumors were found to accumulate ^125^I and they sustained this for 6 h, in contrary with our in vitro result of rapid radioiodine efflux from CNE-2-hNIS cells. This difference can be attributed to the significant re-uptake of radionuclide in vivo, which is contrary to the radioiodine-free medium used in vitro. In our study, ^99m^Tc imaging showed that the experimental group tumors accumulated ^99m^Tc, leading to clear scintigraphic visualization through SPECT, whereas the control tumor was not visualized. Using ^99m^Tc-pertechnetate and SPECT, hNIS gene expression can be monitored both noninvasively and quantitatively.

In this study, ^131^I inhibited CNE-2-hNIS cell proliferation and induced apoptosis in a time- and dose-dependent manner. Our data indicate that a sufficiently high dose of radiation was achieved in CNE-2-hNIS cells and it caused cell death at a dose that spared CNE-2 cells, which were unable to trap ^131^I. Because of the encouraging in vitro cell findings, studies were continued in the in vivo settings. We detected apoptosis and proliferation after ^131^I treatment in vivo. TUNEL, an in-situ cell death detection kit, can preferentially label broken DNA, which generates during the process of apoptosis [23]. Capase-3 can cleave proteins in cells and trigger final apoptotic process. The enhancement in caspase-3 activity therefore is associated with the increase in apoptosis [24]. Ki-67 is a nuclear protein, expressing in all phases of cellular proliferation, not in the G0 phase, which is known as the immunohistochemistry marker for assessing cell proliferation [25]. In our study, TUNEL assay of apoptosis and the caspase-3 positive rate was significantly higher, and the Ki-67 labelling index was lower in the experimental group than in the control group at day 6 and 12 of ^131^I treatment. Our results indicate that ^131^I inhibits proliferation and induces apoptosis in NPC hNIS-transfected cells.

Although the post-treatment tumor volume of the experimental group was slightly larger in size in our study, the apoptosis and proliferation pathology markers showed that tumor apoptosis increased and proliferation rate decreased in the early days after ^131^I treatment. Therefore, we selected DW-MRI to evaluate the early response to ^131^I treatment. DW-MRI for the evaluation of early treatment response offers a lot of promise [26–29]. Usually, DWI could detect this change within a week after treatment [28]. Our results showed that the ADC values increased in the first three days after ^131^I therapy, and reached a peak at day 6 and 12, with a maximum rate of 0.28% ± 0.11% and 0.49% ± 0.19%, respectively.

Changes in the ADC value after cancer treatment are associated with many factors. Our study results showed that ADC values peaked at day 6 and 12 after treatment and that TUNEL assay and caspase-3 immunohistochemistry at both these time points showed that the rate of apoptosis was highest in both periods. ADC values after treatment, apoptosis rate detected by TUNEL assay, and caspace-3 expression were positively correlated (r = 0.72, P < 0.05; r = 0.65, P < 0.05, respectively). The changes in ADC values of mice xenografts after treatment were related with tumor cell apoptosis induced by ^131^I treatment.

In addition, tumor cell density is another important factor of water cell diffusion in tumor tissues [30]. High cellular density is related to a low ADC value, demonstrating that the mobility of water protons is impaired. The ADC values were related to necrotic tissue with inherent diffusion of water protons, which was due to the loss of cell membrane integrity. DWI indeed reflected earlier changes in histology at cellular and subcellular scales. The changes in ADC values of NPC xenografts after ^131^I treatment were also related with cell proliferation.

Treatment with ^131^I therapy can result in increasing water diffusion in tissues by inhibiting tumor cell proliferation and inducing tumor cell apoptosis, which can be detected as an increase in the mean diffusion value of the tumor. Subsequently increasing ADC values after therapy suggest that the treatment was effective. Therefore, DW-MRI could assess the efficacy of ^131^I therapy if there were no changes in the tumor volume after treatment. It is a non-invasive method for early detection of tumor response to treatment after targeted radionuclide therapy.

To further investigate the response of the hNIS-transfected NPC cells after ^131^I therapy, we decided to detect apoptosis-related proteins. Our study showed that P53 and activated Caspase-3 expression was increased in NPC cells after ^131^I therapy, while the expression of Bcl-2 was decreased, especially in cells transfected with hNIS gene. In our study, Survivin expression also increased after ^131^I treatment, and some cells may be considered as radiation resistant after ^131^I exposure. Therefore, we hypothesized that the function of Survivin in tumor cells was blocked or inhibited during ^131^I treatment. Therefore, if we block the ability of Survivin to inhibit apoptosis and reduce the radiation resistance developed during ^131^I treatment, it will enhance the sensitivity of tumor cells to radiation and help us achieve a more effective tumor treatment.

In conclusion, we present the successful treatment of NPC with radioiodide after hNIS transfection. Using ^131^I treatment, a striking cytotoxic effect of proliferation inhibition and apoptosis induction on NPC hNIS-transfected cells was observed in vitro and vivo, which has significant potential for the treatment of NPC. DW-MRI was used for the first time to assess the efficacy of early ^131^I therapy in NPC.
